# Effect of Chemical Order in the Structural Stability and Physicochemical Properties of B_12_N_12_ Fullerenes

**DOI:** 10.1038/s41598-019-52981-1

**Published:** 2019-11-11

**Authors:** Alejandro Escobedo-Morales, Lorenzo Tepech-Carrillo, Alejandro Bautista-Hernández, José Humberto Camacho-García, Diego Cortes-Arriagada, Ernesto Chigo-Anota

**Affiliations:** 1 Facultad de Ingeniería Química, Benemérita Universidad Autónoma de Puebla, Av. San Claudio y 18 Sur, C.P. 72570, Ciudad Universitaria, Puebla, Puebla, Mexico; 2 Unidad de Sistemas Biológicos e Innovación Tecnológica, Universidad Autónoma Benito Juárez de Oaxaca, C.P. 68120, Oaxaca de Juárez, Oaxaca, Mexico; 3 Facultad de Ingeniería, Benemérita Universidad Autónoma de Puebla, Av. San Claudio y 18 Sur, C.P. 72570, Ciudad Universitaria, Puebla, Puebla, Mexico; 4 Programa Institucional de Fomento a la Investigación, Desarrollo e Innovación, Universidad Tecnológica Metropolitana, Ignacio Valdivieso 2409, P.O. Box 8940577, San Joaquín, Santiago Chile

**Keywords:** Carbon nanotubes and fullerenes, Computational methods

## Abstract

The effect of chemical order in the structural and physicochemical properties of B_12_N_12_ [4,6]-fullerene (BNF) isomers was evaluated using density functional theory and molecular dynamic calculations. The feasibility to find stable BNF isomers with atomic arrangement other than the well-known octahedral *T*_*h*_-symmetry was explored. In this study, the number of homonuclear bonds in the modeled nanostructures was used as categorical parameter to describe and quantify the degree of structural order. The BNF without homonuclear bonds was identified as the most energetically favorable isomer. However, a variety of BNF arrays departing from *T*_*h*_-symmetry was determined as stable structures also. The calculated vibrational spectra suggest that isomers with chemical disorder can be identified by infrared spectroscopy. In general, formation of homonuclear bonds is possible meanwhile the entropy of the system increases, but at expense of cohesive energy. It is proposed that formation of phase-segregated regions stablishes an apparent limit to the number of homonuclear bonds in stable B_12_N_12_ fullerenes. It was found that formation of homonuclear bonds decreases substantially the chemical hardness of BNF isomers and generates zones with large charge density, which might act as reactive sites. Moreover, chemical disorder endows BNF isomers with a permanent electric dipole moment as large as 3.28 D. The obtained results suggest that by manipulating their chemical order, the interaction of BNF’s with other molecular entities can be controlled, making them potential candidates for drug delivery, catalysis and sensing.

## Introduction

Boron nitride (BN) compound has gained huge attention because its high mechanical hardness, thermochemical stability, and electric and thermal conductivity. In bulk form, it crystallizes in hexagonal, cubic, rhombohedral and monoclinic structures^1^. Additionally, a variety of BN nanostructures has been already synthetized, such as nanosheets, nanowires, nanotubes and fullerenes^2,3^. These nanosized polymorphs have been identified as promising materials for energy storage^4^, catalysis^5^, molecular sensing^6^, tribology^7^, heat transport^8^ and drug delivery^9^. Particularly, for those systems constituted by a few dozens of atoms, important efforts have been done to determine the effect of atomic arrangement in their functional properties. For example, in 1993 Jensen and Toftlund^10^ proposed several B_12_N_12_ conformational isomers based on their carbon counterparts, namely, monocyclic ring, graphite-like sheet and two fullerene structures. From computational *ab initio* calculations at the MP2/DZP level, they determined that among them, that with truncated octahedron symmetry (point group: *T*_*h*_) has the lowest total energy. This [4,6]-fullerene has 14 faces generated by 36 covalent bonds between B and N atoms with *sp*^2^ hybridization. Six faces correspond to 4-membered rings (2B2N) and the other eight to 6-membered rings (3B3N). The B-N bond lengths belong in the range of 1.44–1.48 Å, being the overall size of the nanostructure around 0.4 nm. Several years later, Strout^11^ performed additional calculations using both Hartree-Fock and density functional theory (DFT) in local and gradient-corrected forms. Again, the octahedral *T*_*h*_-symmetry was identified as the most stable arrangement for B_12_N_12_ conformational isomers. In the late 90 s, Golberg *et al*.^12^ reported a method to obtain this kind of nanostructures using electron beam irradiation. Their B/N stoichiometric ratio was confirmed to be ∼1 by electron energy loss spectroscopy (EELS) and predicted octahedral symmetry was verified through high-resolution transmission electron microscopy (HRTEM).

For simplicity reasons, most theoretical models consider materials as defect-free systems, thus periodic and/or symmetrical atomic arrays are frequently used to study their properties. Nonetheless, at temperatures above 0 K, formation of defects debasing symmetry might occur spontaneously meanwhile the contribution to free energy due to entropy exceeds that of enthalpy. Under this condition, a large defect density may lead to substantial discrepancies between theoretical predictions and experimental measurements.

Evidently, the role of chemical order in the physical and chemical properties become more preponderant as the size of the system decreases. For example, it has been demonstrated that the catalytic, optical and magnetic properties of bimetallic nanoparticles depend on whether their components are distributed is such manner to form mixing patterns or phase-separated arrangements^13,14^. In the case of the B_12_N_12_ fullerenes, although the octahedral *T*_*h*_-symmetry has been identified as the more energetically favorable atomic array, to the best of our knowledge, there is no direct evidence that discards formation of stable [4,6]-fullerenes having homonuclear bonds (B-B and N-N) due to swapping B and N sites, i.e., isomers with some degree of chemical disorder. Because deeper understanding of chemical order may contribute to achieve fine-tuning of molecular functions, the aim of this study is to explore from a theoretical perspective the feasibility to find stable B_12_N_12_ [4,6]-fullerene isomers with atomic arrangement other than the octahedral *T*_*h*_-symmetry, if so, quantify the effect of chemical order in their structural and physicochemical properties.

## Models and Computational Methods

### B_12_N_12_ fullerenes models

**Figure 1:**
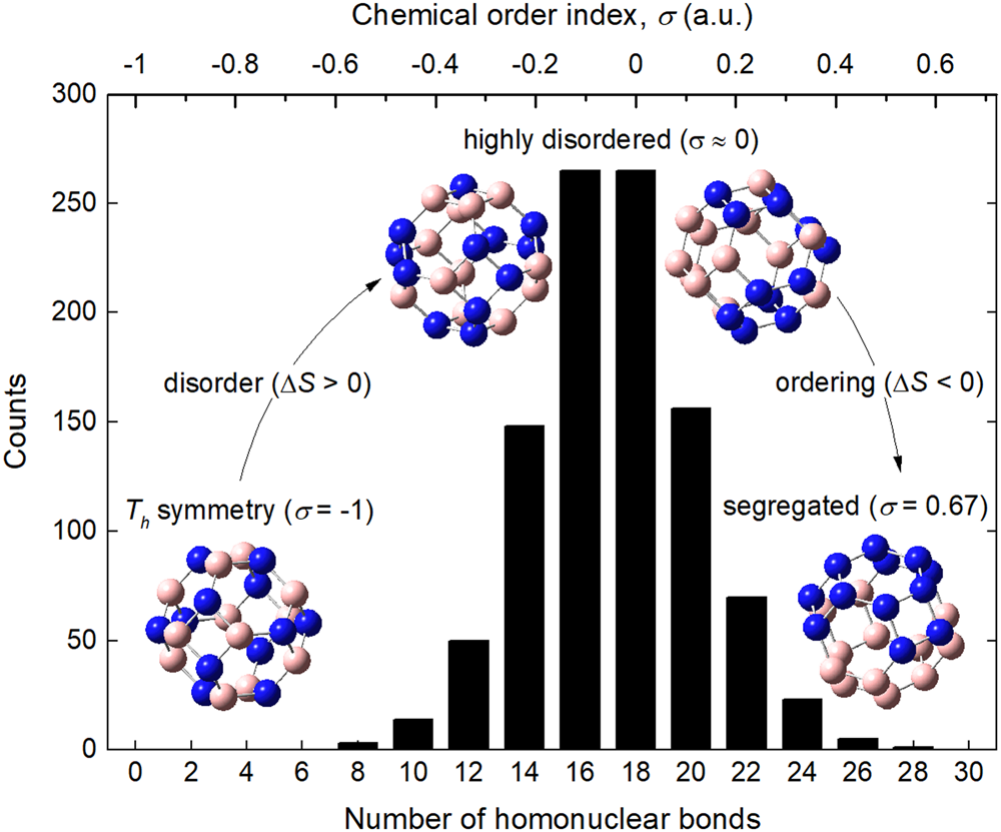
Histogram of the number of homonuclear bonds in a sample of B_12_N_12_ fullerene isomers generated by random (sample size: 1,000).

There is one unique arrangement of atoms to obtain a B_12_N_12_ [4,6]-fullerene (BNF) having *T*_*h*_-symmetry. This B_12_N_12_ conformational isomer has no homonuclear bonds^15^. Hereafter, it is termed as the symmetric BNF isomer. When homonuclear bond restriction is discarded, the number of different arrangements is as large as ∼10^6^. Clearly, a study of such population is intractable without a statistical approach. The methodology to select a representative sample was as follows. Initially, one thousand different BNF initial models (staring points) were generated using a regular truncated octahedron as template (point group: *O*_*h*_; edge length = 1.4 Å). B and N atoms were then placed at the apices randomly. The obtained arrangements were analyzed and organized considering the chemical order index *σ* as categorical attribute (see Fig. 1). This parameter was first introduced by Ducastelle^16,17^ to distinguish between homogeneous (*σ* < 0) and disordered (*σ* ≈ 0) mixtures from that systems with segregated phases (*σ* > 0). In our case, *σ* is also a measure of the number of homonuclear (B-B and N-N) and heteronuclear (B-N) bonds ratio. The chemical order index *σ* for the BNF isomers was defined as:

1 $$\sigma =\frac{{n}_{{\rm{N}} \mbox{-} {\rm{N}}}+{n}_{{\rm{B}} \mbox{-} {\rm{B}}}-{n}_{{\rm{B}} \mbox{-} {\rm{N}}}}{{n}_{{\rm{N}} \mbox{-} {\rm{N}}}+{n}_{{\rm{B}} \mbox{-} {\rm{B}}}+{n}_{{\rm{B}} \mbox{-} {\rm{N}}}},$$

where, *n*_B-N_, *n*_B-B_ and *n*_N-N_ are the number of B-N, B-B and N-N bonds, respectively. Since $\sum n=36$, *n*_B-B_ = *n*_N-N_ = (36 − *n*_B-N_)/2, and defining *n*_homobonds_ = *n*_B-B_ + *n*_N-N_, Eq. (1) can be rewritten as:

2 $$\sigma =\frac{{n}_{{\rm{homobonds}}}}{18}-1.$$

After, an appropriate number of isomers were randomly selected from each category to obtain a distribution that resembles that of the parent sample. Because models laying at the distribution tails have almost negligible probability to be generated through random process (*σ* = −1, 0.67), the sample set was completed by adding them manually. Using this methodology, the initial BNF isomers set was constituted by 50 initial models distributed in 15 categories {*x*, *σ*, *n*} (*x*: number of homonuclear bonds; *σ*: chemical order index; *n*: number of individuals): {0, −1, 1}, {4, −0.78, 2}, {6, −0.67, 2}, {8, −0.56, 2}, {10, −0.44, 3}, {12, −0.33, 3}, {14, −0.22, 5}, {16, −0.11, 8}, {18, 0, 8}, {20, 0.11, 5}, {22, 0.22, 3}, {24, 0.33, 3}, {26, 0.44, 2}, {28, 0.56, 2} and {30, 0.67, 1}. For visualizing the whole set of BNF models please see Supplementary Table S1.

### Computational methods

The structural and physicochemical properties of the BNF isomers were studied by means of first principles calculations based on density functional theory (DFT) within the generalized gradient approximation (GGA). For this purpose, all**-**electron calculations were performed using the HSEh1PBE hybrid functional developed by Perdew, Burke and Ernzerhof^18,19^ and the 6**–**311 g(d,p) basis set proposed by Pople *et al*.^20^ as implemented in the GAUSSIAN**–**09 package^21^. All the systems were considered as neutral in charge and having multiplicity *M* = 1 (*M = *2*S*_*T*_ + 1 = 1, *S*_*T*_ = total spin). Initially, an ultrafine integration grid and stringent convergence criterion (10^−8^ hartrees) were set for the total energy calculations. Then, atomic relaxation was conducted by setting the force convergence threshold at 10^−6^ hartrees bohr^−1^.

The structural stability of the optimized models was verified by calculating their vibrational spectra within the harmonic approximation; non-imaginary frequency was used as stability criterion. Additionally, the kinetic stability of selected optimized isomers was further evaluated at room temperature by calculating propagation of nuclear centers and electron density through *ab-initio* molecular dynamic (MD) calculations using the atom density matrix propagation method (ADMP)^22–24^ as implemented in the GAUSSIAN**–**16 software^25^. For this purpose, the potential was determined “on-the-fly” at the HSE1PBE/6-31 G(d,p) level of theory. MD-ADMP calculations were conducted with a step time of 0.2 fs, and intervals of 2.0 ps were considered for statistical analyses. The control of temperature was attained by velocity scaling at each step, and equations of motion were solved through the Verlet velocity algorithm^26^. The cohesive energy (*E*_coh_) for all isomers was determined as the average difference of energy per atom before and after getting bounded:

3 $${E}_{{\rm{coh}}}=\frac{12({E}_{{\rm{B}}}+{E}_{{\rm{N}}})-{E}_{{\rm{tot}}}}{24},$$

where *E*_B_ and *E*_N_ are the energy of non-interacting boron and nitrogen atoms, respectively, and *E*_tot_ is the total energy of the stable fullerene isomer (gas phase). Further characterization of the modeled systems was addressed by calculating the highest occupied molecular orbital (HOMO) energy *ε*_HOMO_, the lowest unoccupied molecular orbital (LUMO) energy *ε*_LUMO_, HOMO-LUMO gap energy *ε*_HOMO-LUMO_, chemical hardness *η*, Mulliken electronegativity *χ*, dipole moment *p*, and molecular electrostatic potential (MEP) isosurfaces. According to Parr *et al*.^27^ and Zhan *et al*.^28^, *η* equals to *ε*_LUMO_ − *ε*_HOMO_, and *χ* = −(*ε*_LUMO_ + *ε*_HOMO_)/2, being the negative of the chemical potential *μ* (*χ* = −*μ*). Finally, the effect of chemical order in the charge distribution within the BNF isomers was studied by calculating *p* and MEP isosurfaces^29^.

## Results and Discussion

### Total and cohesive energy

**Figure 2:**
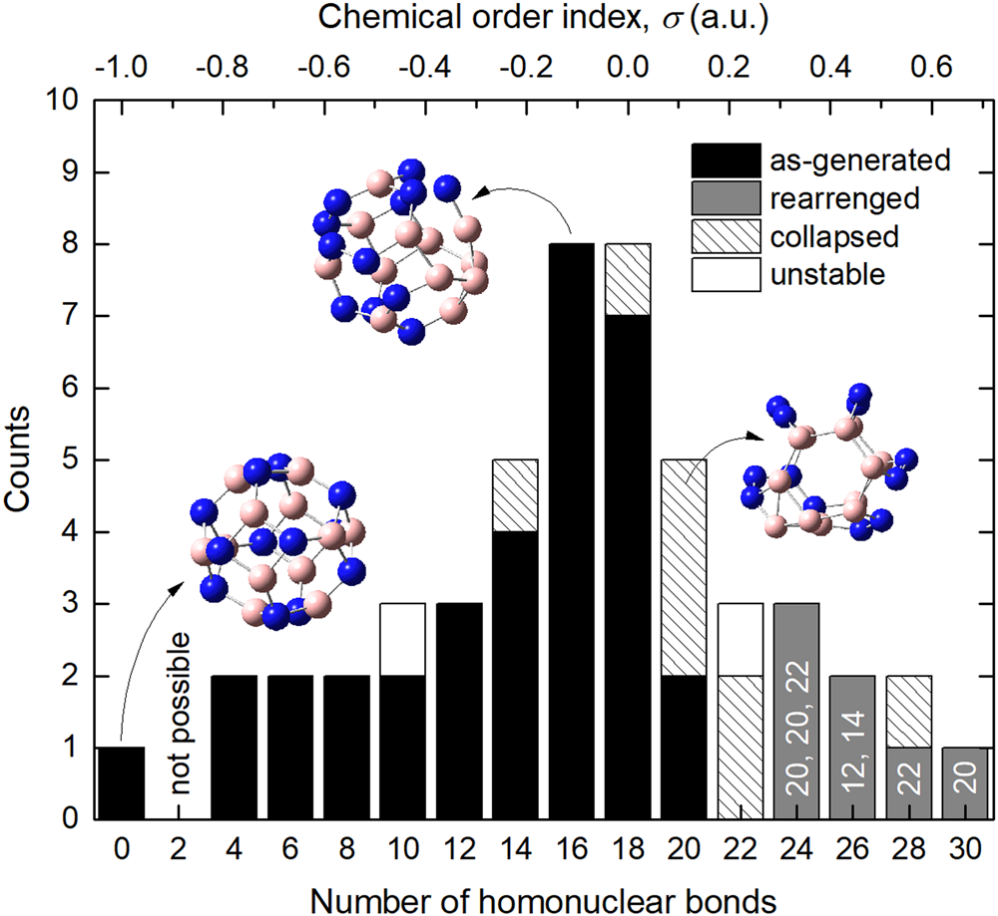
Histogram of the number of homonuclear bonds in a sample of B_12_N_12_ fullerene isomers after geometry optimization (sample size: 50). The labels in the gray bars indicate the final number of homonuclear bonds in those rearranged fullerenes.

Figure 2 shows the histogram of the number of homonuclear bonds of the BNF isomers after geometry optimization. As can be noted, the original sample distribution changed. Among the 50 isomers, 33 fullerenes did not modify their initial number of homonuclear bonds (as-generated), 7 reduced it (rearranged), 8 could not be classified as fullerene-like structures (collapsed) and 2 were identified as unstable configurations. The analysis of physicochemical properties was then realized by considering only those models (40 isomers) which complaint with the stability criterion, remain as Euler (XY)_12_ polytope and whose B-N, B-B and N-N bond lengths are within the range of ±10% the average value.

A comparison of the number of homonuclear bonds before and after geometry optimization suggests that the minimum-energy path gets more biased as the number of homonuclear bonds increases. The latter can be interpreted as absence of local minima of the potential energy surface near the initial arrangements with *σ* ≥ 0.33. Conversely, most of the optimized isomers with *σ* ≤ 0 have closed-shell structure and equal number of homonuclear bonds as the initial ones. Existence of several local minima implies that chemical disorder in B_12_N_12_ fullerenes does not necessary lead the system to an unstable state.

**Figure 3:**
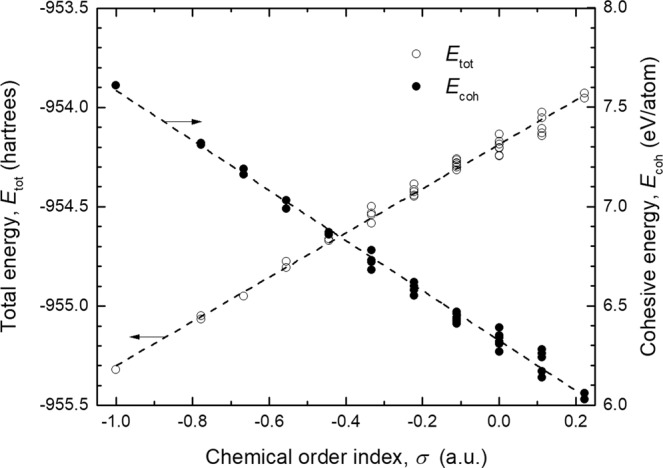
Dependence of the total (*E*_tot_) and cohesive energy (*E*_coh_) with chemical order index (*σ*) of stable B_12_N_12_ fullerene isomers.

Figure 3 presents the calculated total and cohesive energy of the different stable BNF isomers. The lowest total energy corresponds to the symmetric isomer (−955.32011 hartrees). The calculated value matches with that previously reported by Wu *et al*.^30^ (−956.18558 hartrees) using the B3LYP functional along with the correlation-consistent double-zeta (cc-pVDZ) basis set. Additionally, Shevlin *et al*.^31^ estimated the cohesive energy of this fullerene in 7.30 eV/atom using a Perdew–Burke–Ernzerhof (PBE) functional, in agreement with the obtained value of 7.61 eV/atom. In the case of stable isomers with some degree of structural disorder, the total energy increases linearly with chemical order index. This behavior indicates that the energy difference among isomers resides mostly in the homonuclear bonds rather than structural stress. Since two homonuclear bonds (one B-B and one N-N) are formed at expense of two heteronuclear bonds, the total energy of the BNF must change in steps nearly to (*E*_B-B_ + *E*_N-N_) − 2*E*_B-N_ as *σ* increases. Under this reasoning, the magnitude of such discrete changes is estimated in 0.12 hartrees (75.3 kcal mol^−1^) each. It agrees with the reported dissociation enthalpies of B-N (89.4 kcal mol^−1^), B-B (68.4 kcal mol^−1^) and N-N (37.3 kcal mol^−1^) bonds^32^. Because thermal energy at room temperature is around 0.59 kcal mol^−1^ (gas phase), spontaneous reduction of the chemical order index in disordered BNF’s is not expected without an additional energy supply.

Lack of stable isomers with phase-segregated regions (numerous homonuclear bonds) and large total energy (low cohesive energy) can be understood from thermodynamic considerations. Assuming that the energy to generate homonuclear bonds were supplied as heat, the entropy of the system, *S*, should increase progressively as *σ* approaches to zero (Δ*S* > 0). It agrees with the microscopic interpretation of entropy introduced by Boltzmann^33^, since the category with the largest number of different BNF arrangements corresponds to *σ* ≈ 0 (see Fig. 1). However, if the number of homonuclear bonds is increased further (*σ* > 0) chemical segregation is favored. These atomic arrays might be related to non-equilibrium states because the total energy of the system rises, but at the same time, it gets ordered, reducing its entropy; note that the number of different possible configurations decreases sharply as *σ* gets more positive. The previous arguments stablish an apparent limit to the number of homonuclear bonds in stable B_12_N_12_ fullerenes. In this sense, it is proposed that formation of homonuclear bonds can occur meanwhile the entropy of the system increases, but at expense of its cohesive energy.

### Physicochemical properties

**Figure 4:**
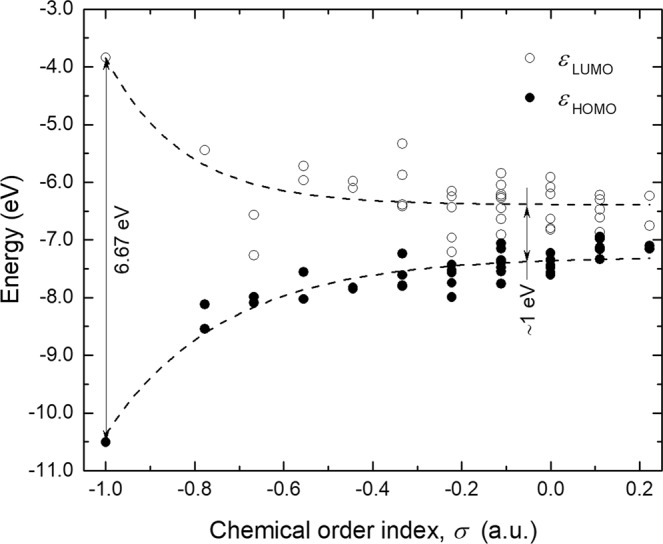
Lowest unoccupied molecular orbital energy (*ε*_LUMO_) and highest occupied molecular orbital energy (*ε*_HOMO_) of B_12_N_12_ fullerene isomers categorized by their chemical order index (*σ*).

**Figure 5:**
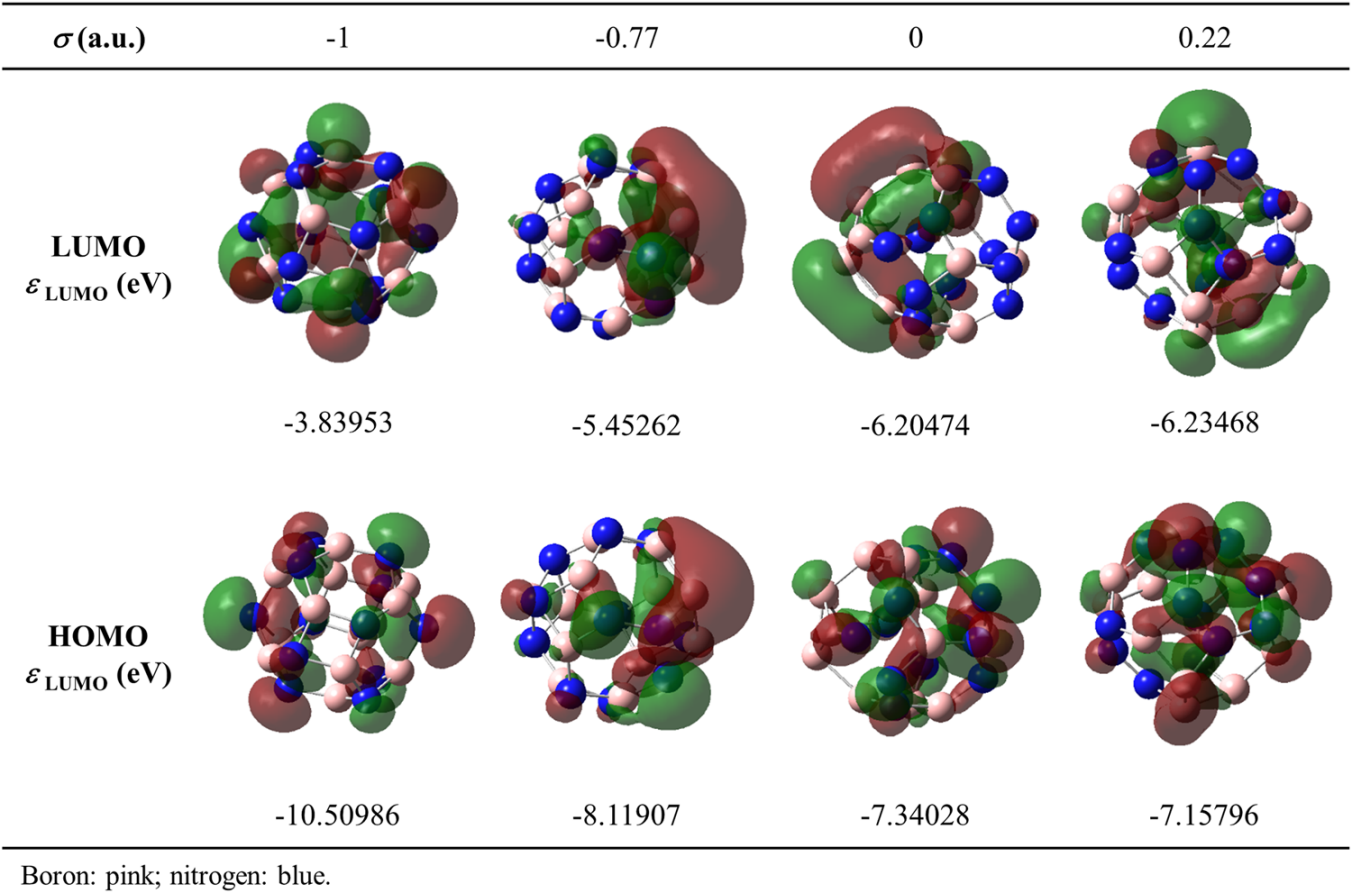
Highest occupied molecular orbital (HOMO), lowest unoccupied molecular orbital (LUMO) and their respective energies (*ε*_HOMO_ and *ε*_LUMO_) of representative B_12_N_12_ fullerene isomers with different chemical order index (*σ*).

Figure 4 presents the dependence of the *ε*_HOMO_ and *ε*_LUMO_ with *σ* of the different stable BNF isomers. In general, the frontier orbitals show an opposite behavior, in such a way that their energies approach as the chemical order index increases. The *ε*_HOMO-LUMO_ of the symmetric fullerene was calculated to be 6.67 eV, but it decays exponentiality up to an asymptotic value of ∼1 eV as *σ* increases. In agreement, Soltani *et al*.^34^ and Baei^35^ reported the *ε*_HOMO-LUMO_ of the symmetric B_12_N_12_ fullerene in 7.20 and 6.85 eV, respectively. These values indicate that this nanostructure behaves as an insulating material. Interestingly, formation of just a few homonuclear bonds induces an insulator-semiconductor transition. Since the *ε*_HOMO-LUMO_ of BNF isomers with *σ* > −1 readily trends to a similar value, it is proposed that the features of the HOMO and LUMO in disordered B_12_N_12_ fullerenes is determined mainly by the states associated to homonuclear bonds, rather than some specific atomic array. Figure 5 shows the HOMO and LUMO of four representative BNF isomers with different chemical order index. As expected, the frontier orbitals of the symmetric BNF are evenly distributed throughout the structure, whereas they displace towards homonuclear bonds in those isomers with σ > −1.

**Figure 6:**
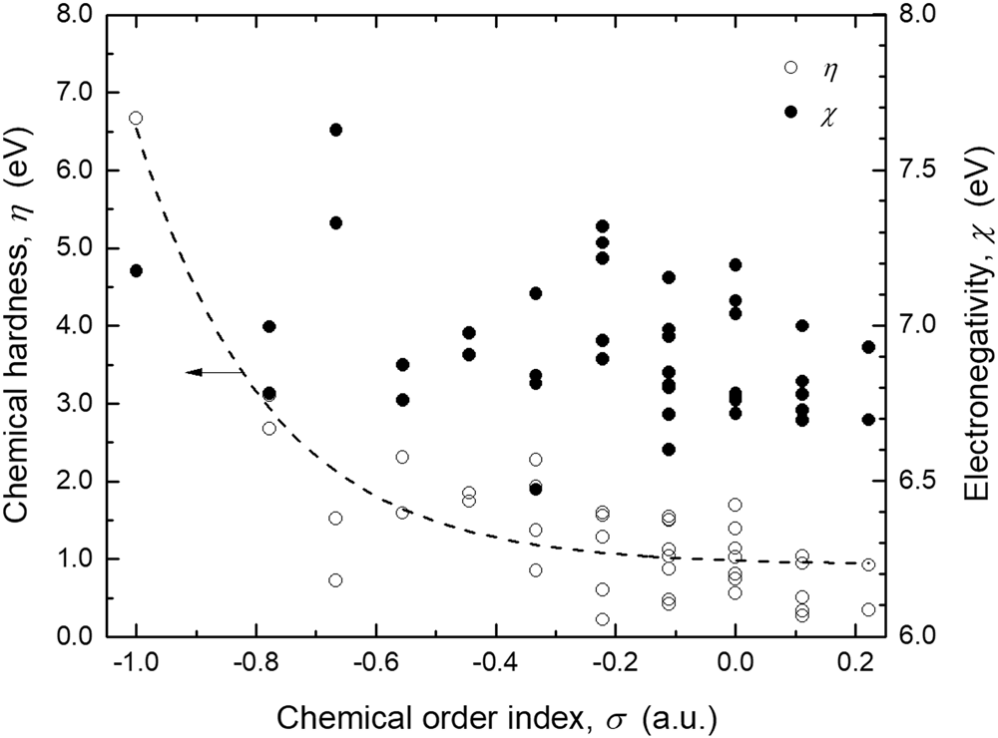
Chemical hardness (*η*) and electronegativity (*χ*) of the B_12_N_12_ fullerene isomers categorized by chemical order index (*σ*).

An insight of the chemical behavior of the BNF isomers was elucidated from analyzing their chemical hardness (*η*) and electronegativity (*χ*) (see Fig. 6). As can be noted, *η* trends to decrease exponentially with *σ*. In the case of the symmetric BNF, it was calculated to be 6.67 eV; the highest among the stable isomers and comparable to that of water (>7 eV)^27^. This result indicates that this fullerene does not tend to form covalent bonds with other molecules. Nonetheless, chemical hardness decreases considerably due to formation of homonuclear bonds, and even lower values than that of molecular potassium (*η*_K2_ < 2 eV)^28^ are calculated for isomers with intermediate chemical order index (*σ* = −0.22; *η* = 0.21 eV). It follows that, chemical disorder leads readily B_12_N_12_ fullerenes to be more liable to interact with their surroundings.

As discussed above, the HOMO and LUMO displace towards homonuclear bonds generated by chemical disorder, forming potential reactive sites. This, along with formation of B- or N-rich zones might trigger fullerenes to react with nearby chemical species or analogous BNF isomers. It holds specially for those isomers with large chemical order index (σ > 0). Seifert *et al*.^36^ proposed that heteronuclear bonds would be sufficient to give BNF a local energy minimum, but also a pathway to dissociation by elimination of molecular nitrogen.

Conversely to chemical hardness, the electronegativity of the BNF isomers does not have an evident dependence with *σ*. For the symmetric fullerene, it was calculated to be 7.17 eV. The obtained *χ* values for the rest of isomers belong in the range of 6.47–7.62 eV (*χ*_avg_ = 6.92 eV; *s* = 0.22 eV). Therefore, the capacity to attract electrons by B_12_N_12_ fullerenes does not decrease due to chemical disorder.

**Figure 7:**
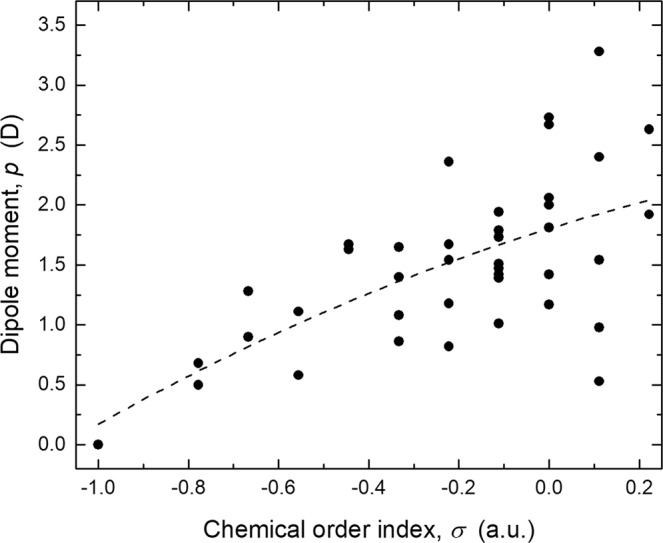
Dipole moment (*p*) of the stable B_12_N_12_ fullerene isomers categorized by chemical order index (*σ*).

Certainly, chemical interaction among molecular entities can be described in terms of their chemical hardness and electronegativity. Nonetheless, their interaction is mediated by the force filed generated by the spatial charge distribution within the system. In this regard, the magnitude of dipole moment (*p*) is a convenient observable, not just for describing the charge distribution, but to elucidate the interaction among chemical species through electrostatic and/or dipole-dipole attraction^37^. Figure 7 presents the magnitude of the electric dipole moment for the different BNF isomers. In the case of the symmetric fullerene, its *T*_*h*_-symmetry entails a null permanent dipole moment. Therefore, its interaction with other non-polar entities must be weak. Nonetheless, adsorption of low-electronegative sorbates through charge transfer mechanism or interaction with high polar molecules by induced dipole attraction still might be possible.

As described previously, chemical disorder tends to reduce the gap energy between the frontier orbitals and redistributes their charge density. It is because variations in the structural configuration provoke changes of the electronic states. Then, for those BNF isomers having σ > −1, the new electron distribution along with an asymmetric location of B and N nuclei generates a permanent dipole moment. In general, the magnitude of *p* increases with *σ*, and values as high as 3.28 D were calculated. Since, those molecules having large permanent dipole are more easily solubilized in strong polar media, it is proposed that chemical disorder does not just enhance the capability of bare B_12_N_12_ fullerenes as molecular sorbents, but their feasibility to be solubilized in aqueous media.

**Figure 8:**
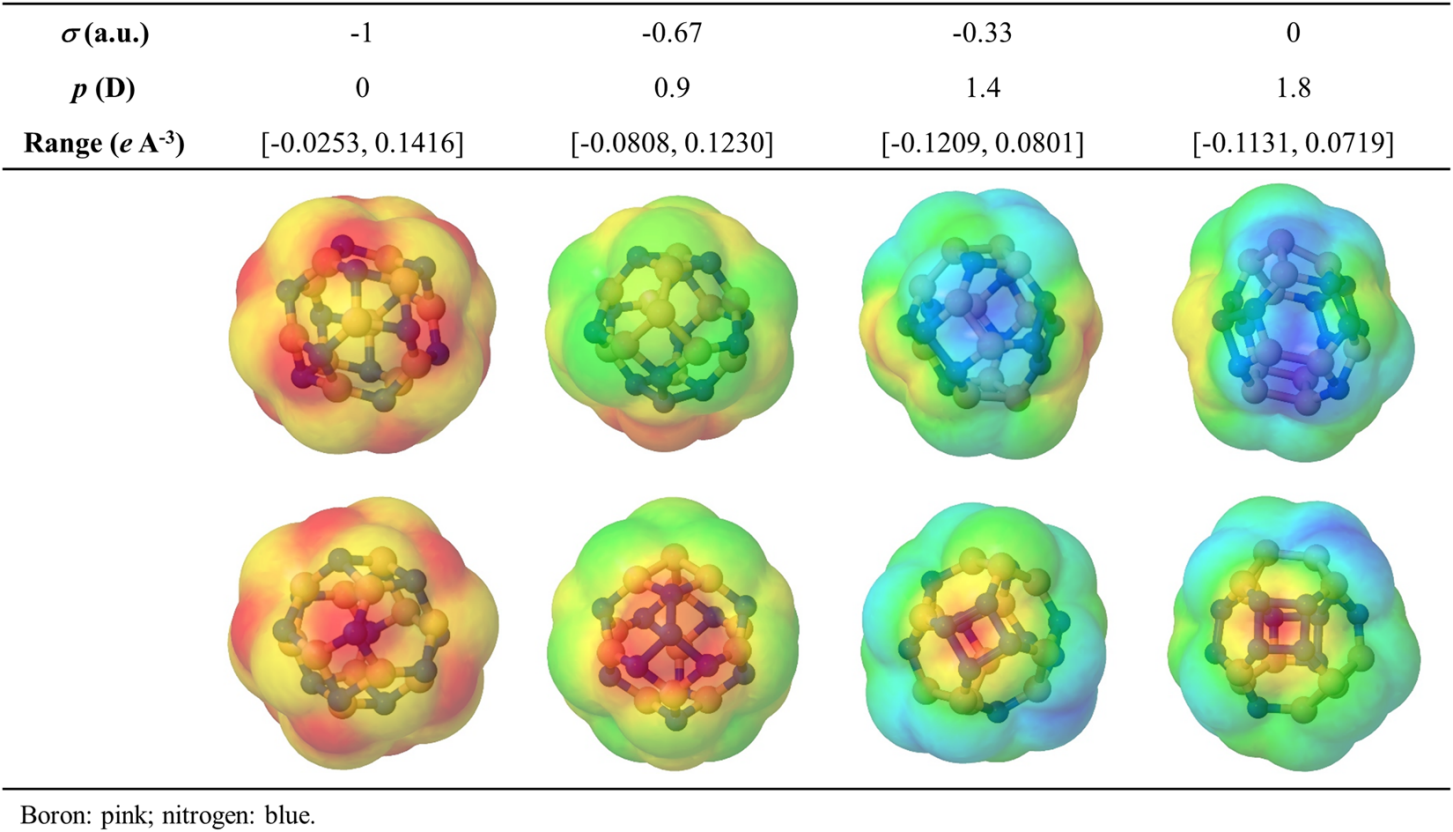
Electric dipole moment (*p*) and molecular electrostatic potential (MEP) isosurfaces showing the largest positive (blue) and negative (red) charged zones of representative B_12_N_12_ fullerene isomers with different chemical order index (*σ*).

Certainly, the magnitude of *p* shows an apparent tendency to increase with chemical order index (i.e., the number of homonuclear bonds). Nonetheless, it is worth noting that for some isomers having the same number of homonuclear bonds, their dipole moment can be six times as large as another analogous isomer. Moreover, the results suggest the range of *p*-values increases in size with *σ*. It could be just because the number of possible atomic configurations growths significantly as *σ* approaches zero. In any case, it is evident that the magnitude of the permanent dipole moment generated by chemical disorder depends on the atomic arrangement. Further detail of its influence in the spatial distribution charge, and thus, the permanent dipole moment, can be obtained by calculating their MEP isosurfaces. Figure 8 shows the MEP’s isosurfaces of representative BNF isomers with different chemical order index. As expected, for the isomer without homonuclear bonds (*σ* = −1), the charge density is symmetrically distributed around its mass center, being the electron density concentrated at the more electronegative nitrogen atoms. However, if two B-N pairs swap their sites (*σ* = −0.67), two analogous mononuclear groups resembling centered triangles are formed. Again, the N-group concentrates the electronic charge, and its position respect to the positive charged zone (B-group) determines the polar character of the fullerene. Since the positive and negative charged zones can be adjacent, but not overlapped, a non-zero dipole moment is a distinctive physicochemical feature of B_12_N_12_ fullerenes with some degree of chemical disorder. In this regard, large *p*-values seem to be related to formation of nitrogen rings (*σ* = −0.33, 0). The latter explains the general relationship between *p* and *σ* observed in Fig. 7, on considering that the probability to form such structural features increases with the number or homonuclear bonds.

**Figure 9:**
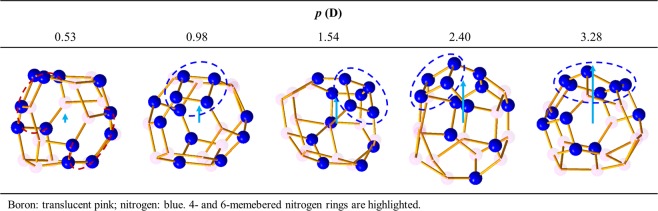
Electric dipole moment (*p*) of B_12_N_12_ fullerene isomers with equal chemical order index (*σ* = 0.11; 20 homonuclear bonds). The arrows represent the relative magnitude and direction of *p*.

The likely correlation between the magnitude of the permanent dipole moment with some structural motif was confirmed by analyzing the atomic arrangement of several isomers with equal chemical order index. Figure 9 presents the dipole moment of the isomer category with *σ* = 0.11 (20 homonuclear bonds). The isomer with the smallest dipole moment is constituted by two incomplete nitrogen rings (1B5N) separated by a boron chain. Thus, the opposite position of these N-groups balances the negative charge around the mass center. Formation of 4-membered nitrogen rings are recognized in those isomers with intermediate dipole moment (*p* = 0.98, 1.54, 2.40 D). In agreement with MEP isosurfaces, this geometrical feature matches with the largest positive charge density, and the resulting *p*-value is determined by its position respect to the largest B-group. Finally, the most polar isomer (*p* = 3.28 D) is characterized by a 6-membered nitrogen ring. Here, segregation of nitrogen atoms facilitates boron counterpart groups form at the opposite hemisphere of the fullerene structure. It follows, *p* increases notoriously.

### Vibrational modes and kinetic stability

**Figure 10:**
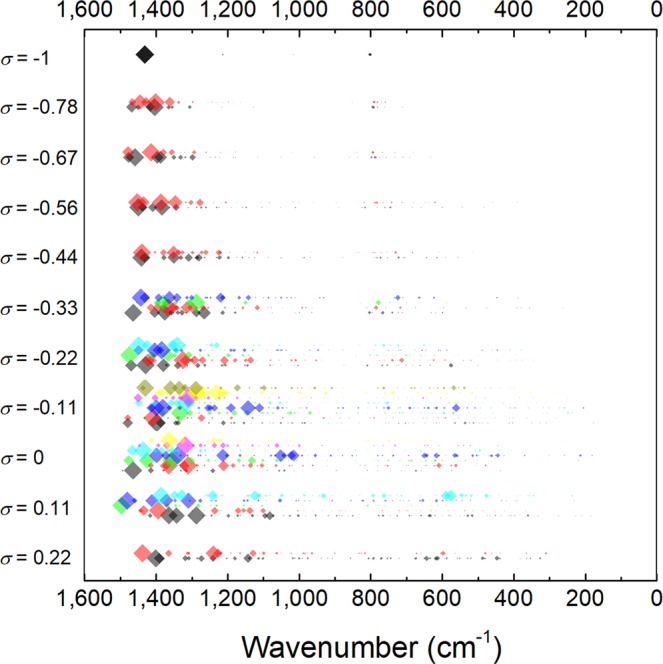
Calculated frequencies and relative absorbances (indicated by diamond size) of infrared bands of B_12_N_12_ fullerene isomers categorized by their chemical order index (*σ*).

**Figure 11:**
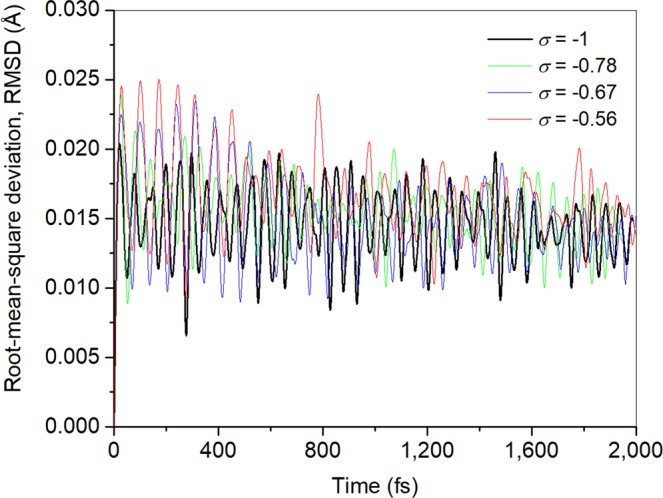
Root-mean-square deviation (RMSD) of the atomic coordinates in thermalized BNF fullerenes isomers (300 K) with different chemical order index (*σ*) as compared with the corresponding ground state geometry (0 K). Step time: 0.2 fs; number of steps: 10,000.

**Figure 12:**
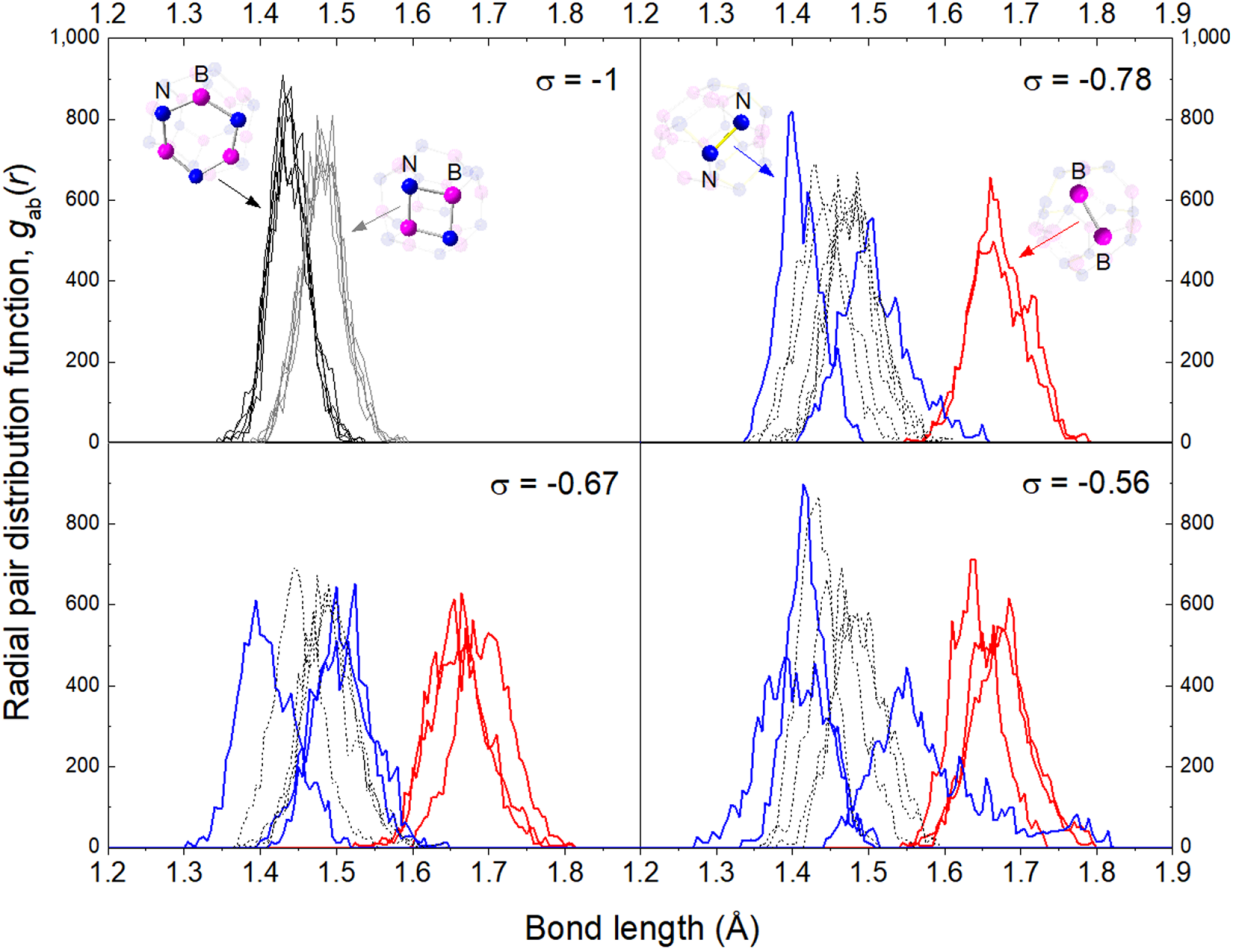
Radial pair distribution function, *g*_ab_(*r*), of bond lengths for the most stable ordered (*σ* = −1) and disordered (*σ* = −0.78, −0.67, −0.56) B_12_N_12_ fullerene isomers at 300 K. Step time: 0.2 fs; number of steps: 10,000.

Since vibrational modes of materials are sensitive to the atomic arrangement, the corresponding vibrational spectrum can be considered as the fingerprint of each structure. In this sense, structural disorder in B_12_N_12_ fullerenes could be identified by analyzing their Raman and/or infrared (IR) spectrum. Figure 10 presents the calculated vibrational frequencies of the different BNF isomers (IR spectra of representative fullerenes are presented in Supplementary Fig. S1). The strongest infrared band of the symmetric BNF is located at 1,432 cm^−1^. It is attributed to symmetric stretching mode of N-B-N groups belonging to 4-memebered rings. Additionally, a weak band associated to wagging mode of 1B3N and 3B1N groups appears at 803 cm^−1^ ^36^. In the case of isomers with chemical disorder, the latter results in an increasing number of infrared-active vibrational modes. It is proposed that this feature might be exploited through infrared spectroscopy as indicator of a fraction of isomers having some degree of structural disorder. In analogy to the symmetric BNF isomer, two groups of infrared bands are distinguished. The first corresponds to strong high-frequency modes (1,500–1,000 cm^−1^), whereas the second to weaker low-frequency modes (800–200 cm^−1^). A systematic smearing towards lower frequencies are observed in both groups as *σ* increases. Several authors have suggested that low-frequency vibrational modes may be correlated with irreversible structural rearrangements of disordered systems near to the threshold of mechanical stability^38,39^. Thus, these transitions are associated to systems settled at local minima with low-potential energy barriers. In order to identify possible structural rearrangements of BNF isomers with chemical disorder towards *T*_*h*_ symmetry or unstable states, the kinetic stability of the most stable ordered (*σ* = −1) and disordered (*σ* = −0.78, −0.67, −0.56) isomers was explored through *ab-initio* molecular dynamic trajectories at 300 K. Figure 11 shows the root-mean-square deviation (RMSD) of the atomic coordinates in thermalized systems as compared with the corresponding ground state geometry (0 K). As can be seen, at the very early stage of calculations, molecular motions get stable and no considerable change in the atomic positions is observed regardless kinetic energy increases. The results show that the ground state geometry of the analyzed BNF isomers remain almost unchanged, with average atomic displacements as large as 0.025 Å. Therefore, MD-ADMP calculations indicate that these isomers are highly stable and even distinguishable at room temperature. This behavior is attributed to potential energy barriers are high enough that avoid isomerization reactions once they are formed. Additionally, the radial pair distribution functions *g*_ab_(*r*) of heteronuclear and homonuclear bonds were calculated under dynamic conditions (see Fig. 12). In the case of the symmetric fullerene, the *g*_ab_(*r*) functions show a slight difference in the characteristic B−N bond length. It is determined that bonds constituting four-membered rings have lengths mainly retained in the range 1.40–1.60 Å, and those of six-membered rings belong in range 1.35–1.50 Å. When chemical disorder is present, the bond distances fall in the ranges 1.30–1.60 Å, 1.35–1.60 Å and 1.60–1.80 Å for B-N, N-N and B-B bonds, respectively. As can be noted, the dispersion of *g*_ab_(*r*) functions increases with the number of homonuclear bonds. It suggests that kinetic stability of BNF isomers progressively decreases with chemical disorder, in agreement with the calculated cohesive energies.

## Conclusions

The effect of chemical order in the structural and physicochemical properties of B_12_N_12_ [4,6]-fullerene (BNF) isomers was studied from a theoretical perspective using density functional theory and molecular dynamic calculations. In agreement with previous reports, the octahedral *T*_*h*_-symmetry was identified as the most energetically favorable atomic arrangement; its cohesive energy was calculated to be around 7.6 eV/atom. However, a variety of stable BNF isomers having homonuclear bonds generated by chemical disorder were found also. In general, formation of homonuclear bonds is possible meanwhile the entropy of the system increases, although it occurs at expense of cohesive energy. Whereas the electronegativity of the BNF does not show an evident dependence with chemical order, its chemical hardness decreases considerably due to formation of homonuclear bonds, corresponding to the sites with large charge density. Conversely to the symmetric fullerene, the isomers with some degree of chemical disorder have an intrinsic electric dipole, which is favored by formation of large N- and B-rich moieties (4- or 6-membered rings). These changes in the physicochemical properties could make BNF’s with chemical disorder more liable to interact with their surroundings and be solubilized in polar media. Finally, it is proposed that using infrared spectroscopy is possible to identify whether a fraction of stoichiometric BNF present structural disorder by analyzing the number of bands in the range of 1,500–1,000 cm^−1^.

## Supplementary information

Supplementary information
