# Supplementary material for: Effect of Chemical Order in the Structural Stability and Physicochemical Properties of B12N12 Fullerenes
Source: Sci Rep. 2019 Nov 11;9:16521. doi: 10.1038/s41598-019-52981-1 (PMC6848210; doi:10.1038/s41598-019-52981-1)
Supplement: Supplementary file 1 — Supplementary information [file 41598_2019_52981_MOESM1_ESM.pdf]

SUPPLEMENTARY INFORMATION

Effect of Chemical Order in the Structural Stability and Physicochemical Properties of $B_{12}N_{12}$ Fullerenes

A. Escobedo-Morales, L. Tepech-Carrillo, A. Bautista-Hernández, J. H. Camacho-García, D. Cortes-Arriagada and E. Chigo-Anota

1. Initial and Optimized Models

Table S1. Initial and geometry optimized $B_{12}N_{12}$ fullerene models. Chemical order index before (σ_{ini}) and after (σ_{opt}) optimization as well as calculated total energy E_{tot} (in hartrees) are shown for each isomer [σ_{ini}, σ_{opt}, E_{tot}].

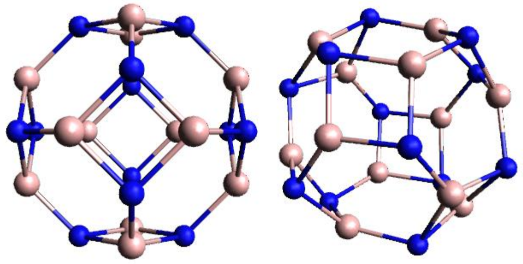

[-1, -1, -955.32011]

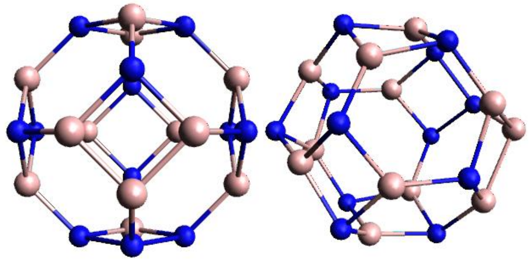

[-0.78, -0.78, -955.06574]

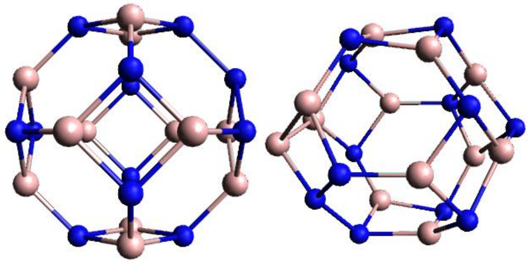

[-0.78, -0.78, -955.05047]

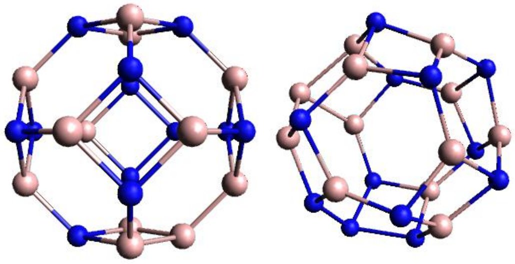

[-0.67, -0.67, -954.94996]

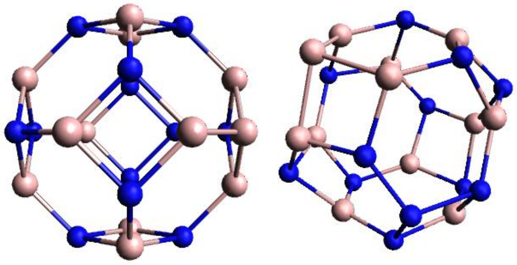

[-0.67, -0.67, -954.94996]

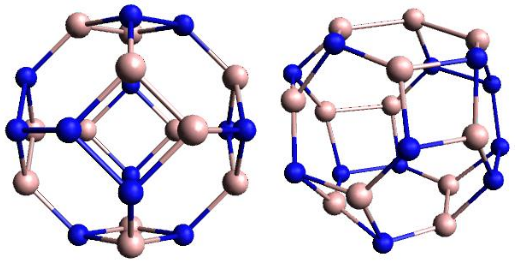

[-0.56, -0.56, -954.7744]

Table S1. (continuation).

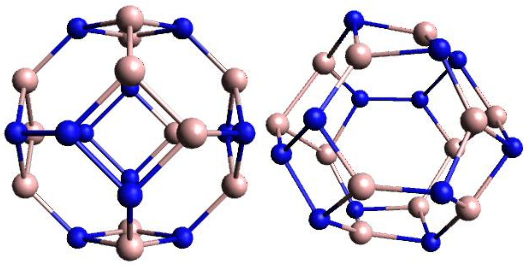

[-0.56, -0.56, -954.80681]

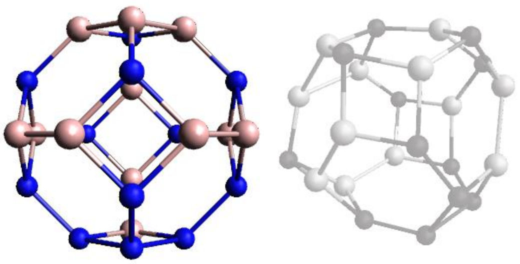

[-0.44, **UNSTABLE**]

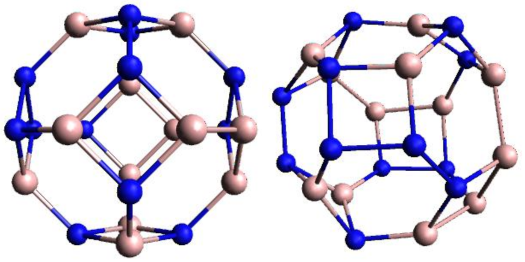

[-0.44, -0.44, -954.66104]

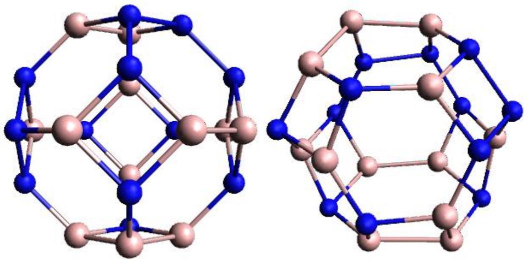

[-0.44, -0.44, -954.67086]

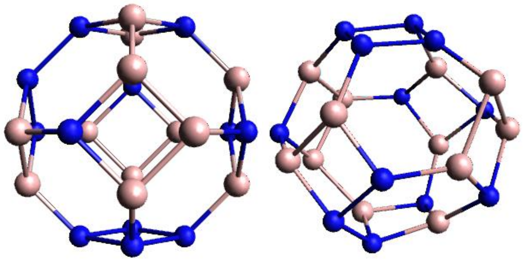

[-0.33, -0.33, -954.5319]

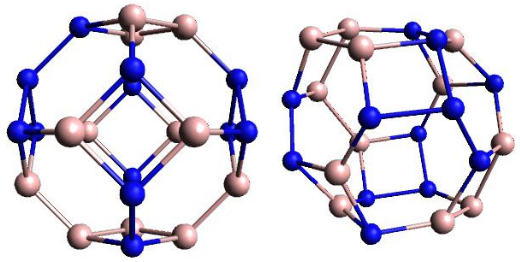

[-0.33, -0.33, -954.58413]

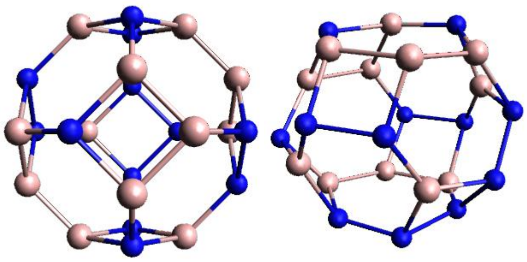

[-0.33, -0.33, -954.4992]

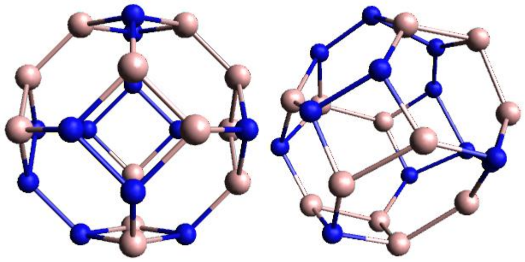

[-0.22, -0.22, -954.42608]

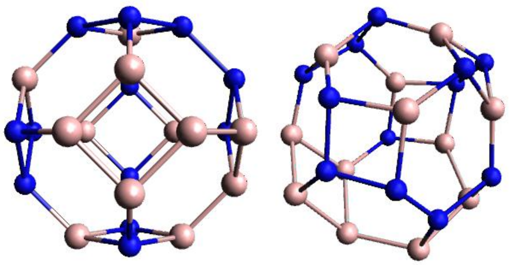

[-0.22, -0.22, -954.41428]

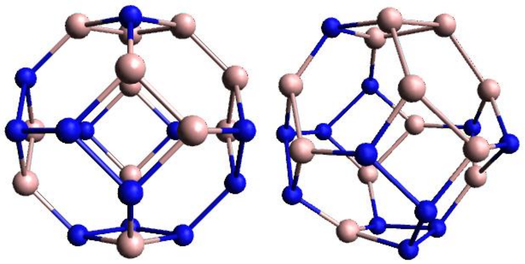

[-0.22, -0.22, -954.38538]

Table S1. (continuation).

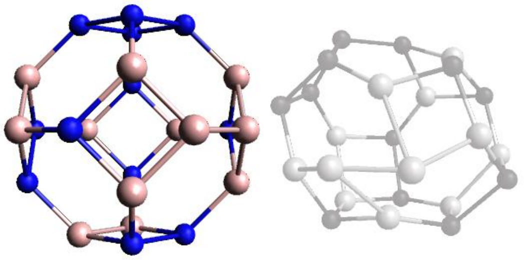

[-0.22, **COLLAPSED**]

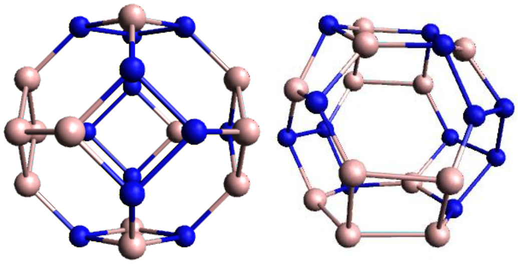

[-0.22, -0.22, -954.44713]

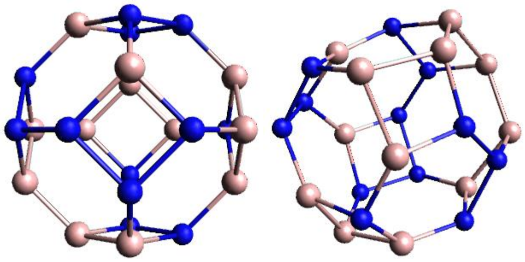

[-0.11, -0.11, -954.27794]

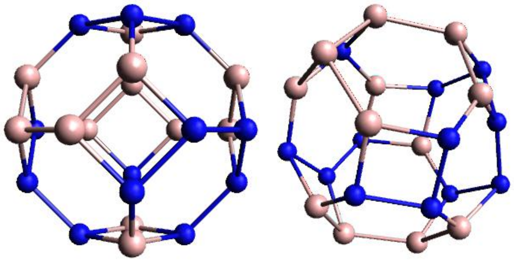

[-0.11, -0.11, -954.26615]

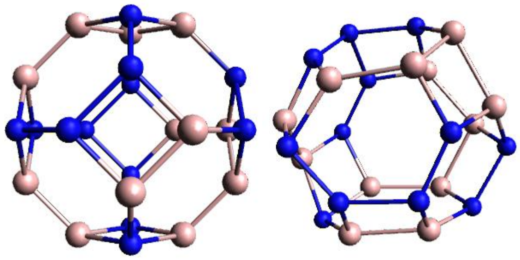

[-0.11, -0.11, -954.25832]

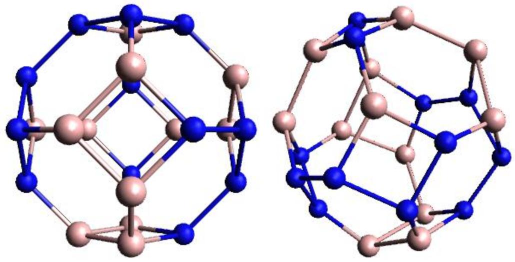

[-0.11, -0.11, -954.31625]

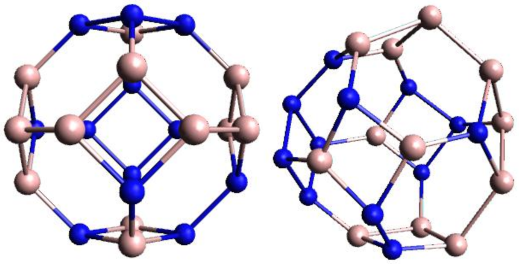

[-0.11, -0.11, -954.3009]

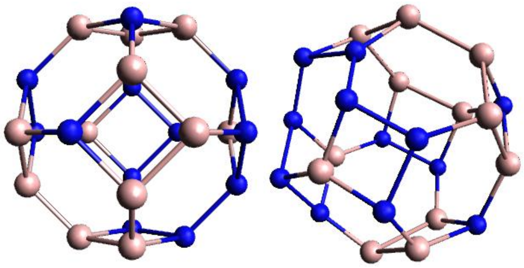

[-0.11, -0.11, -954.28169]

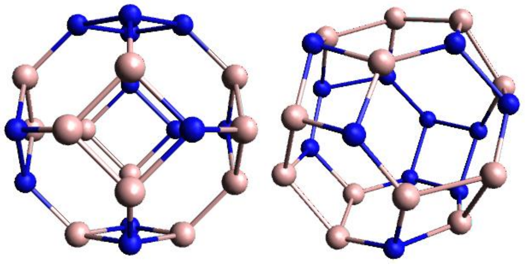

[-0.11, -0.11, -954.28863]

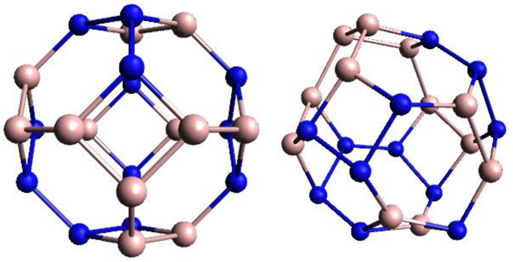

[-0.11, -0.11, -954.28161]

Table S1. (continuation).

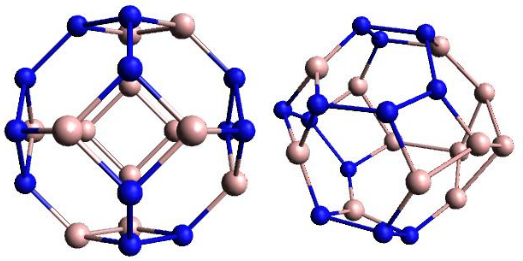

[0, 0, -954.24317]

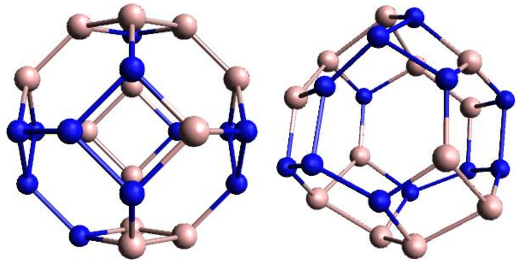

[0, 0, -954.20628]

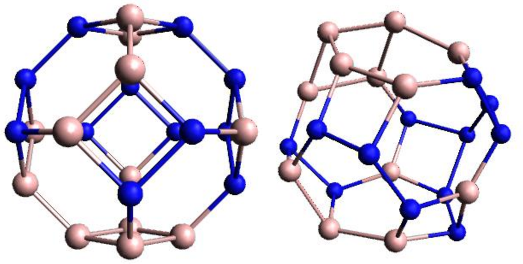

[0, 0, -954.2019]

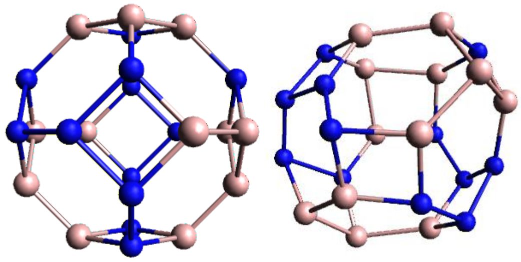

[0, 0, -954.13545]

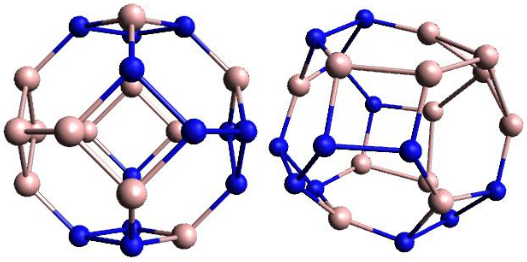

[0, 0, -954.24491]

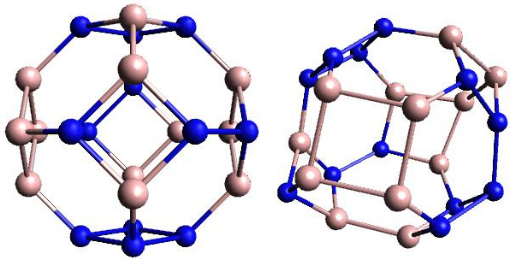

[0, 0, -954.18485]

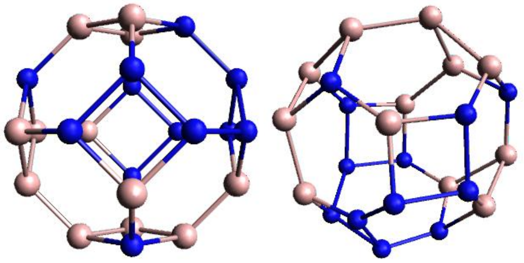

[0, 0, -954.17136]

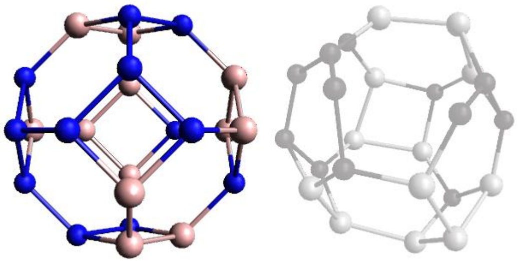

[0, **COLLAPSED**]

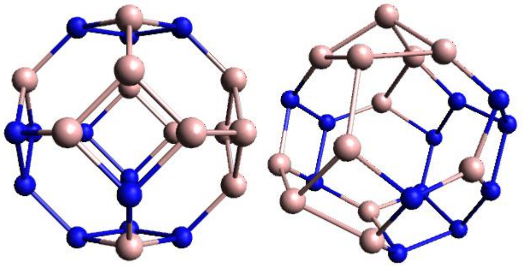

[0.11, 0.11, -954.13002]

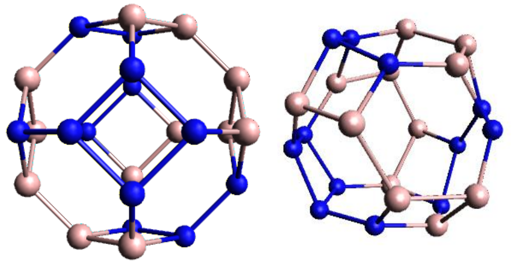

[0.11, 0.11, -954.02341]

Table S1. (continuation).

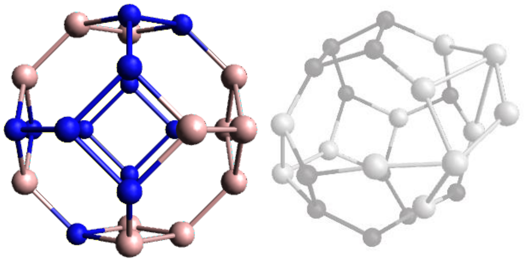

[0.11, **COLLAPSED**]

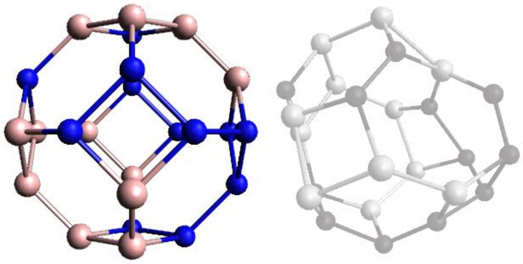

[0.11, **COLLAPSED**]

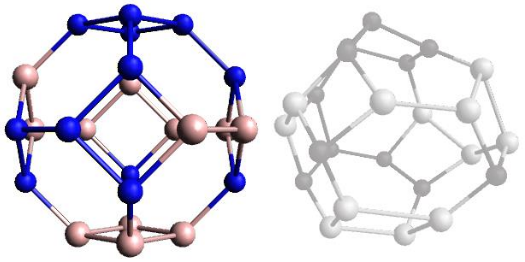

[0.11, **COLLAPSED**]

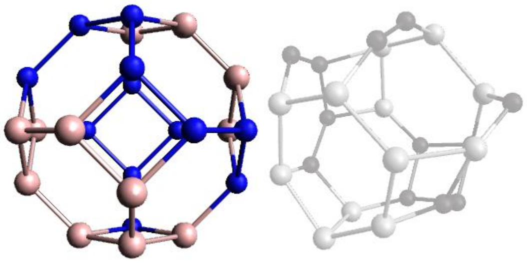

[0.22, **COLLAPSED**]

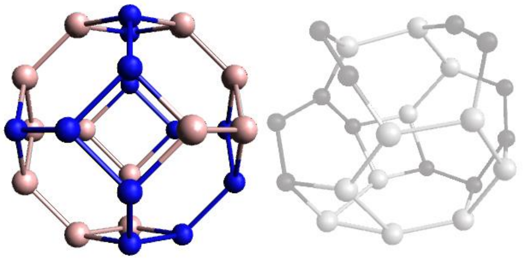

[0.22, **COLLAPSED**]

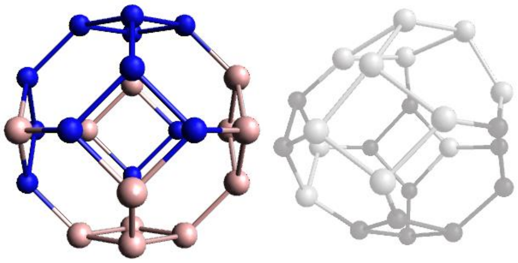

[0.22, **UNSTABLE**]

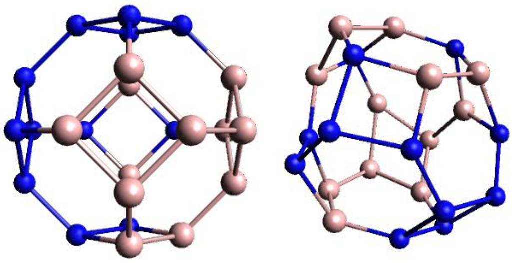

[0.33, 0.11, -954.14225]

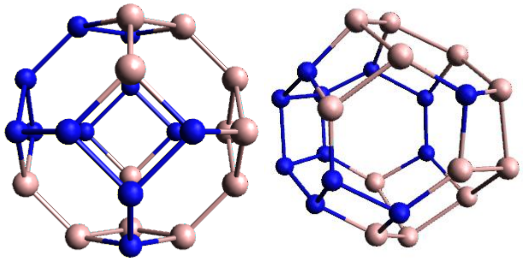

[0.33, 0.22, -953.95319]

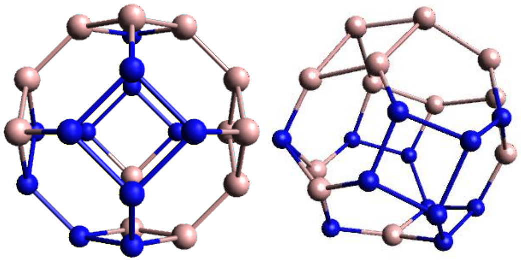

[0.33, 0.11, -954.10673]

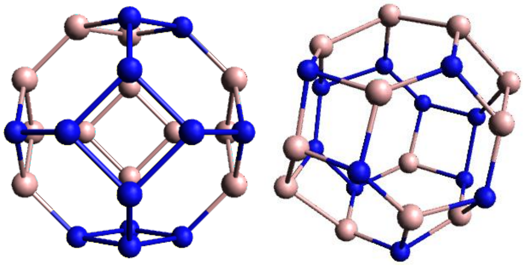

[0.44, -0.33, -954.54139]

Table S1. (continuation).

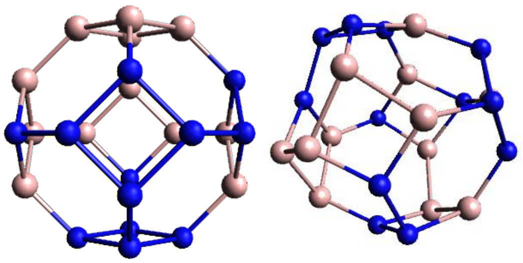

[0.44, -0.22, -954.44176]

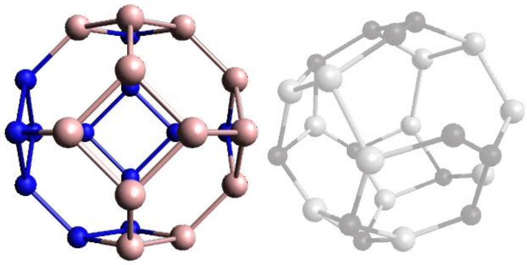

[0.56, **COLLAPSED**]

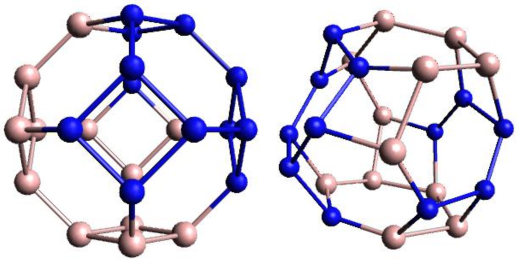

[0.56, 0.22, -953.92803]

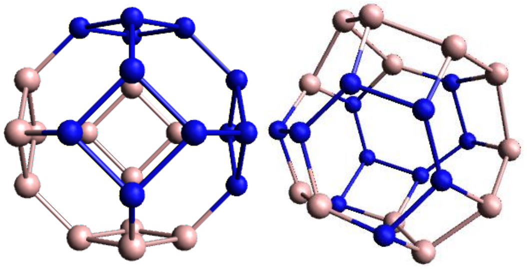

[0.67, 0.11, -954.05214]

2. Atomic Coordinates

2.1. Truncated octahedron template

Atomic coordinates of the regular truncated octahedron (point group: O_h; edge length = 1.4 Å) used as template to generate the set of initial $B_{12}N_{12}$ fullerene models.

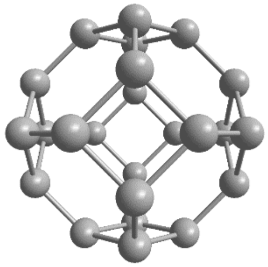

```
structure = X12Y12_template
number_of_homonuclear_bonds = -
chemical_order_index = -
status = -
units = angstroms
symbol    x    y    z
-    1    2    0
-    0    2    1
-    0    1    2
-    1    0    2
-    2    0    1
-    2    1    0
-    -1   0    -2
-    0    -1   -2
-    0    -2   -1
-    -1   -2   0
-    -2   -1   0
-    -2   0    -1
-    0    2    -1
-    -1   2    0
-    -2   1    0
-    -2   0    1
-    -1   0    2
-    0    -1   2
-    0    -2   1
-    1    -2   0
-    2    -1   0
-    2    0    -1
-    1    0    -2
-    0    1    -2
```

2.2. $B_{12}N_{12}$ fullerene structures

Atomic coordinates of the initial and geometry optimized $B_{12}N_{12}$ fullerene models with chemical order index σ = -1, -0.78, -0.67, -0.56.

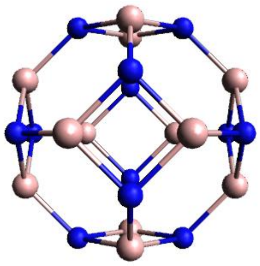

```
structure = B12N12_0HOM_1
number_of_homonuclear_bonds = 0
chemical_order_index = -1
status = initial
units = angstroms
symbol    x    y    z
N    1    2    0
B    0    2    1
N    0    1    2
B    1    0    2
N    2    0    1
B    2    1    0
B    -1   0    -2
N    0    -1   -2
B    0    -2   -1
N    -1   -2   0
B    -2   -1   0
N    -2   0    -1
B    0    2    -1
N    -1   2    0
B    -2   1    0
N    -2   0    1
B    -1   0    2
N    0    -1   2
B    0    -2   1
N    1    -2   0
B    2    -1   0
N    2    0    -1
B    1    0    -2
N    0    1    -2
```

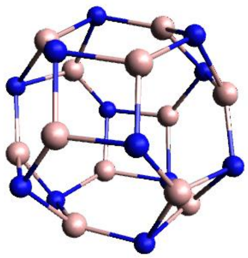

```
structure = B12N12_0HOM_1_optimized
number_of_homonuclear_bonds = 0
chemical_order_index = -1
status = optimized
units = angstroms
symbol    x    y    z
N    -0.0897319     -2.3747764     0.3338179
N    -1.7911323     1.2154554 1.036064
N    0.7044301 1.5728803 1.66942
N    2.211385  0.8728266 -0.3294281
N    1.7911371 -1.2154594     -1.0360672
N    0.4766644 0.6670669 -2.2542723
N    -2.2113902     -0.8728296     0.3294289
N    -1.6400458     0.8486208 -1.5324472
N    -0.4766646     -0.6670668     2.2542731
N    1.6400456 -0.8486198     1.532447
N    0.0897328 2.3747804 -0.3338171
B    -0.5332583     1.8993505 0.92339
B    -1.5863304     -0.182685 1.481818
B    0.3584318 -1.5874364     1.44785
B    0.5332602 -1.8993485     -0.9233902
B    2.1107667 -0.4991691     0.2213099
B    1.2665948 1.7455309 0.3096779
B    -0.3584319     1.5874384 -1.4478492
B    -2.1107668     0.4991661 -0.2213101
B    -1.266596 -1.7455299     -0.3096781
B    -0.715103 -0.1877297     -2.0471912
B    1.5863302 0.182683  -1.4818182
N    -0.7044302     -1.5728793     -1.6694192
B    0.7151028 0.1877307 2.0471911
```

structure = B12N12_4HOM_2
number_of_homonuclear_bonds = 4
chemical_order_index = -0.78
status: initial
units = angstroms

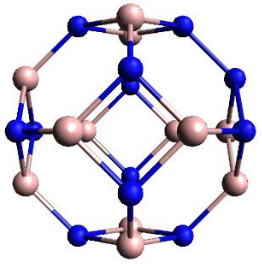

symbol	x	y	z
N	1	2	0
N	0	2	1
N	0	1	2
B	1	0	2
N	2	0	1
B	2	1	0
B	-1	0	-2
N	0	-1	-2
B	0	-2	-1
N	-1	-2	0
B	-2	-1	0
N	-2	0	-1
B	0	2	-1
B	-1	2	0
B	-2	1	0
N	-2	0	1
B	-1	0	2
N	0	-1	2
B	0	-2	1
N	1	-2	0
B	2	-1	0
N	2	0	-1
B	1	0	-2
N	0	1	-2

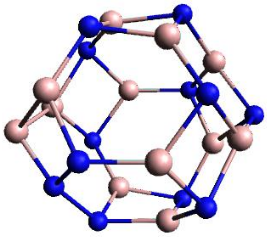

```
structure = B12N12_4HOM_2_optimized
number_of_homonuclear_bonds = 4
chemical_order_index = -0.78
status: optimized
units = angstroms
symbol	x	y	z
N	-0.1915427	1.3804957	-1.7929812
N	1.1707791	1.0008191	1.8404311
N	-1.3724287	0.2188003	1.9878051
N	-1.7688468	-1.6321836	0.213854
N	-1.146572	-1.0127722	-1.8520942
N	0.6591838	-2.2673387	-0.4834061
N	1.8691759	1.6050821	-0.1977431
N	2.2089754	-0.9004508	0.383956
B	-0.7164868	2.4005341	0.457333
N	-2.1635912	0.8577969	-0.3673991
N	0.1790007	-1.388608	1.9139531
B	0.1025744	0.0806246	2.1502021
B	0.8722638	2.0401831	0.808006
N	-1.0722837	1.8033776	-0.7817561
B	-0.1259517	-0.053231	-2.1105422
B	-1.9525736	-0.5071788	-0.7337481
B	-1.1932352	-1.1374225	1.4364421
B	1.060293	-1.7157008	0.831412
B	2.0131029	0.469923	0.746072
B	1.2149213	1.1147175	-1.3641952
B	1.5839294	-1.1918261	-0.9283551
B	-0.7055736	-1.9309612	-0.7729901
N	1.3372677	-0.2304548	-1.9727012
B	-1.7460287	1.2219488	1.031678
```

structure = B12N12_6HOM_2
number_of_homonuclear_bonds = 6
chemical_order_index = -0.67
status = initial
units = angstroms

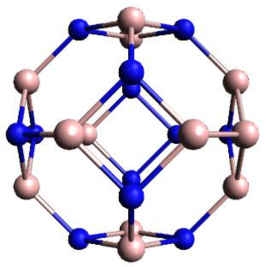

symbol	x	y	z
B	1	2	0
B	0	2	1
N	0	1	2
B	1	0	2
N	2	0	1
B	2	1	0
B	-1	0	-2
N	0	-1	-2
B	0	-2	-1
N	-1	-2	0
B	-2	-1	0
N	-2	0	-1
B	0	2	-1
N	-1	2	0
N	-2	1	0
N	-2	0	1
B	-1	0	2
N	0	-1	2
B	0	-2	1
N	1	-2	0
B	2	-1	0
N	2	0	-1
B	1	0	-2
N	0	1	-2

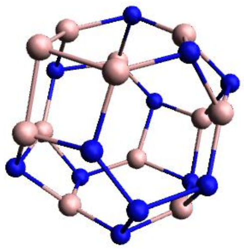

structure = B12N12_6HOM_2_optimized
number_of_homonuclear_bonds = 6
chemical_order_index = -0.67
status = optimized
units = angstroms
symbol x y z
N -0.6683909 -2.203959 0.9169011
N -1.6329992 1.0631609 -1.1896961
N -0.6684241 2.2039813 0.9168301
N 1.7381622 1.120447 1.2757931
N 1.7381842 -1.1203872 1.2758331
N 2.2059332 0.0000011 -1.0274021
N -1.6329939 -1.0632292 -1.1896701
N 0.4153621 -0.0000348 -2.3797172
N -1.9415521 -0.0000006 0.9524201
B -0.448255 0.0000418 2.6147112
N 0.6910088 2.1006449 -0.868541
B -0.7324076 1.9420154 -0.508738
N -2.3378931 -0.0000239 -0.394896
B -1.237302 -1.1038979 1.6667581
B 0.7794607 -1.9687935 0.6134781
B 1.1456011 0.000039 2.0493062
B 0.7794306 1.9688258 0.6134171
B 1.2065868 0.9512809 -1.5573051
B -0.92371 -0.0000384 -1.8818121
B -0.7323814 -1.9420379 -0.508679
B 1.2066025 -0.9513122 -1.5572741
B 2.1572932 0.0000212 0.4022781
N 0.6910394 -2.1006594 -0.868474
B -1.2373301 1.1039382 1.6667261

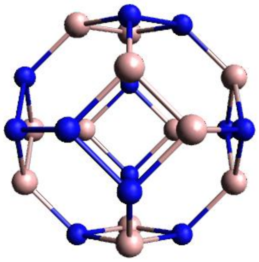

structure = B12N12_8HOM_1
number_of_homonuclear_bonds = 8
chemical_order_index = -0.56
status = initial
units = angstroms

symbol	x	y	z
N	1	2	0
B	0	2	1
N	0	1	2
N	1	0	2
B	2	0	1
B	2	1	0
B	-1	0	-2
N	0	-1	-2
B	0	-2	-1
B	-1	-2	0
B	-2	-1	0
N	-2	0	-1
B	0	2	-1
N	-1	2	0
B	-2	1	0
N	-2	0	1
B	-1	0	2
B	0	-1	2
N	0	-2	1
N	1	-2	0
N	2	-1	0
N	2	0	-1
B	1	0	-2
N	0	1	-2

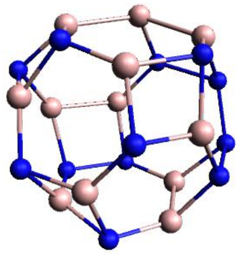

```
structure = B12N12_8HOM_1_optimized
number_of_homonuclear_bonds = 8
chemical_order_index = -0.56
status = optimized
units = angstroms
symbol    x    y    z
N    -0.9227425     1.1709618 -1.7141622
N    -1.471924 -0.1708135     1.8816111
N    -0.5759072     -2.2207484     0.616269
N    1.5492387 -1.5180035     -0.6291091
B    1.407707  0.2712946 -1.7616242
N    2.335349  0.5992649 0.541828
N    -1.9636417     1.6212444 0.626463
B    0.6643825 1.4618164 1.8181641
N    -2.2956639     -0.6606519     -0.5360061
N    -0.6776165     -1.4715723     -1.8542782
N    0.9966046 -1.2126157     1.8517321
B    -0.4967756     -1.2210073     1.7075751
B    -2.1860652     0.1657772 0.625245
B    -1.381061 -0.2005735     -1.6213152
N    0.5906046 1.4364873 -1.7788752
B    0.7344975 -1.2204128     -1.7815842
B    0.8542998 -1.8619528     0.55654
B    1.5996421 0.0744396 1.7561091
B    -1.0240875     1.2343157 1.7202561
B    -1.1373788     1.9918808 -0.4905981
N    1.3179375 1.7769336 0.592775
N    2.3540773 -0.1431801     -0.6549281
B    0.5226763 2.1505414 -0.5142911
B    -1.2886794     -1.7363486     -0.5351241
```

3. Vibrational Modes

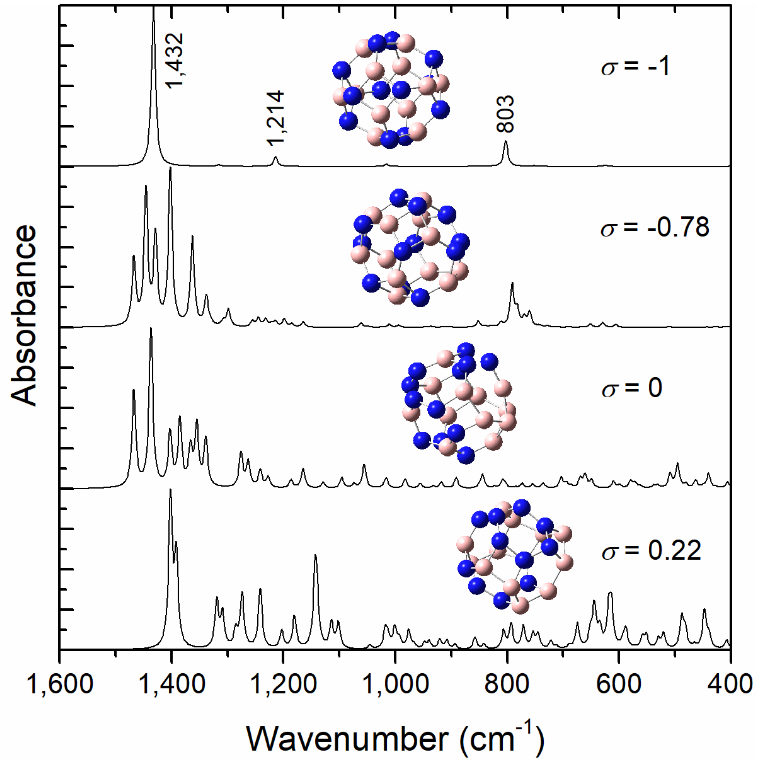

Figure S1. Calculated infrared spectra of representative $B_{12}N_{12}$ fullerene isomers with different chemical order index (σ).

4. Statistical Approach: Sample Size

In order to test whether the original sample size (50 individuals; set A) is representative of the BNF population, a second and independent set of equal number of BNF isomers (set B) was generated and analyzed using the same methodology. A comparison of the physicochemical descriptors between set A and B is shown in Figures S2-S5. As can be seen, the results are similar, and no divergent information is obtained from set B. It demonstrates that both samples are representative of the BNF population and, at the same time, that the selected sample size is large enough to draw general conclusions.

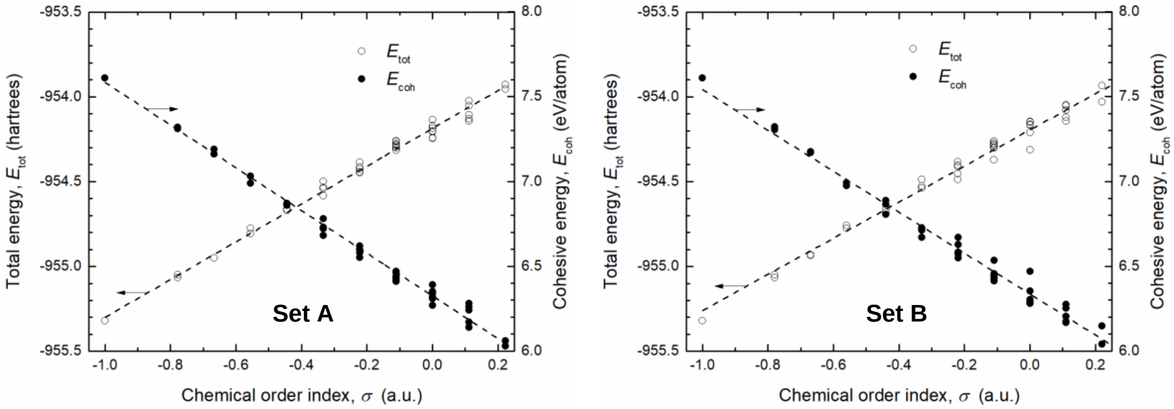

Figure S2. Dependence of the total (E_{tot}) and cohesive energy (E_{coh}) with chemical order index (σ) of stable $B_{12}N_{12}$ fullerene isomers.

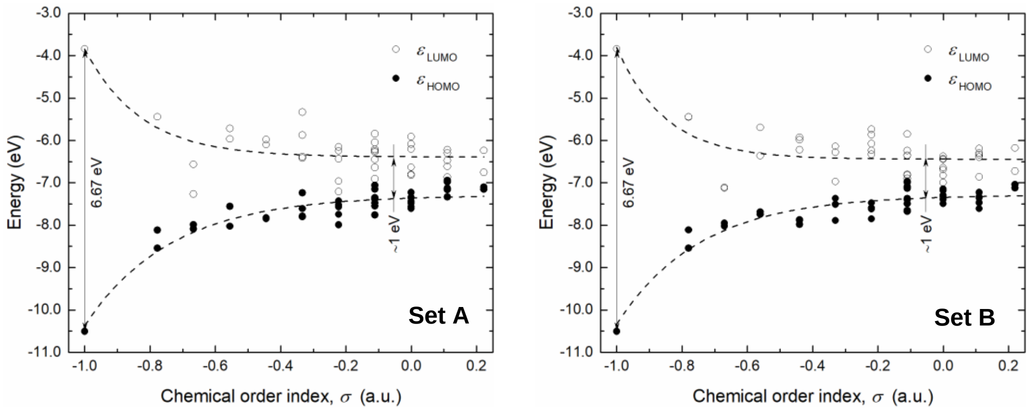

Figure S3. Lowest unoccupied molecular orbital energy (ε_{LUMO}) and highest occupied molecular orbital energy (ε_{HOMO}) of $B_{12}N_{12}$ fullerene isomers categorized by their chemical order index (σ).

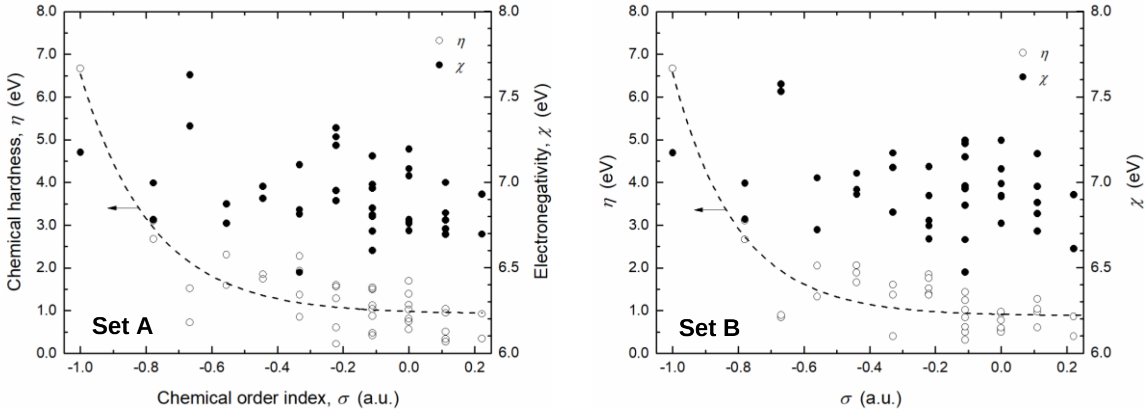

Figure S4. Chemical hardness (η) and electronegativity (χ) of the $B_{12}N_{12}$ fullerene isomers categorized by chemical order index (σ).

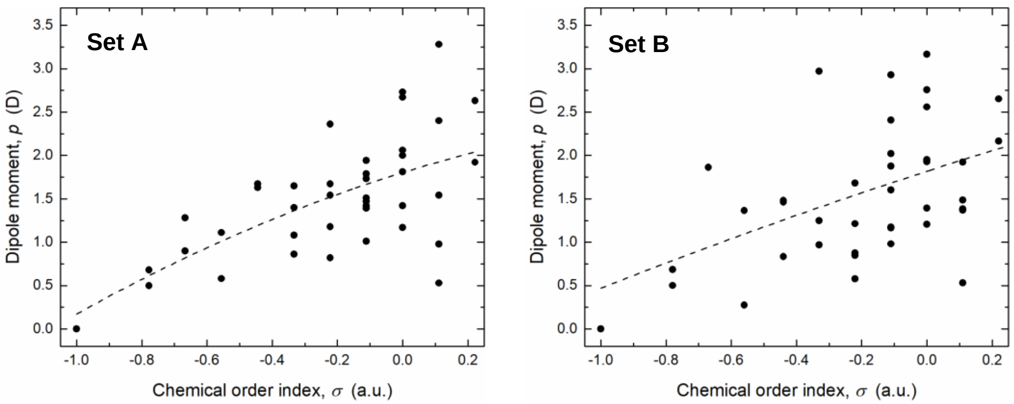

Figure S5. Dipole moment (p) of the stable $B_{12}N_{12}$ fullerene isomers categorized by chemical order index (σ).
